# Self-reported parkinsonian symptoms in the EPIC-Norfolk cohort

**DOI:** 10.1186/1471-2377-5-15

**Published:** 2005-08-24

**Authors:** Lianna S Ishihara, Kay-Tee Khaw, Robert Luben, Sheila Bingham, Ailsa Welch, Nicholas Day, Carol Brayne

**Affiliations:** 1 Department of Public Health and Primary Care, University of Cambridge, Forvie Site, Robinson Way, Cambridge CB2 2SR, UK; 2 Clinical Gerontology Unit, Addenbrookes Hospital, University of Cambridge, Cambridge CB2 2QQ, UK; 3 MRC Dunn Nutrition Unit, Wellcome Trust/MRC Building, Hills Road, Cambridge CB2 2XY, UK

## Abstract

**Background:**

Parkinsonian symptoms have been associated with increased morbidity and mortality. Several studies have reported on the prevalence of signs and symptoms. Symptoms questionnaires can identify potential PD cases for further neurological examination to save resources. They can also provide information about how much of the population reports specific signs and symptoms. The objective of the study was to determine the self-reported prevalence of parkinsonian symptoms from a questionnaire, and to examine their association with age and self-reported Parkinson's disease in a large cohort.

**Methods:**

A cross-sectional study was conducted within a sub-cohort of the EPIC-Norfolk (European Prospective Investigation of Cancer) cohort study.

**Results:**

The prevalence of six self-reported parkinsonian symptoms are reported for 11539 individuals who answered all symptoms questions (62% of sub-cohort): rest tremor (4%), difficulty starting to walk (4%), difficulty getting out of a chair (6%), slower walking (34%), smaller handwriting (micrographia- 9%), and less acute sense of smell (olfactory dysfunction- 9%). The presence of individual symptoms increased with age except for difficulty getting out of a chair.

**Conclusion:**

The results support previous findings that the presence of self-reported parkinsonian symptoms is strongly associated with age and self-reported PD diagnosis. The data also provide information regarding the prevalence of symptoms in a large, younger population of adults than previously reported in the literature.

## Background

Parkinsonian symptoms have been associated with increased morbidity and mortality in Alzheimer's disease and in individuals without dementia [1,2]. In one prospective cohort study, progression in gait disturbance, measured with the UPDRS, was associated with mortality [3]. Individuals with Parkinson's Disease (PD) by definition will have a greater prevalence of symptoms and signs than those without PD, and the age-dependence of parkinsonism predicts that older age groups will have higher proportions of reported signs and symptoms. Several studies have reported the presence of parkinsonian signs and symptoms in elderly individuals without a diagnosis of PD [1,4-11].

The diagnosis of PD is based on clinical signs because there is currently no conclusive diagnostic test available [12]. Clinical characteristics manifest only when the substantia nigral cell loss has reached a threshold of 60 to 80%, and most parkinsonian symptoms and signs are not specific to PD [12-14].

There are four cardinal motor signs of Parkinsonism: resting tremor at 4–6 Hertz, bradykinesia, rigidity, and impaired postural and righting reflexes. Other PD symptoms include: shuffling gait, cogwheel rigidity, unilateral onset, persistent asymmetry in severity, micrographia, masked facies, and retropulsion [15]. Reduced olfactory function has recently been suggested as a feature indicating pre-clinical PD, although it is not specific to PD [16-19].

The purpose of this study was to determine the prevalence of self-reported parkinsonian symptoms within a sub-cohort of EPIC-Norfolk [20]. We report the prevalence of six symptoms by age and PD status.

## Methods

The design and methods of the EPIC-Norfolk cohort study have been described previously [20]. We provide a brief summary of the details regarding the sub-cohort used for the current analyses. The cohort includes men and women who were aged between 45 and 74 years at the time they entered the study. Subjects were recruited through selected General Practitioner (GP) practices in Norfolk, United Kingdom. Invitations were sent to 77 630 individuals and the response rate was 39% (N = 30 447). The main purpose of cohort recruitment was to conduct a prospective study of healthy participants willing to be followed up over time, so the aim was not to recruit a representative population sample. Nevertheless, the EPIC population is similar in age, sex and race to the national sample in the Health Survey for England though with a slightly lower proportion of current smokers.

The population used for studying Parkinsonian symptoms in the EPIC cohort includes only those that completed a second health questionnaire eighteen months after baseline (N = 18 465), when there were specific questions about parkinsonian symptoms. Baseline characteristics are reported for the total cohort, individuals who did not respond to the Parkinson's questionnaire, and those in the current sub-cohort (Table 1).

**Table 1:** Comparison of baseline characteristics between the total EPIC-Norfolk cohort and those completing the 18-month follow-up health questionnaire (current sub-cohort).

Baseline Characteristics	2^nd ^health questionnaire (Sub-cohort)	Non-respondents to the 2^nd ^health questionnaire	Total Cohort
Total Number	18465	11980	30445
Age (Years), Mean ± SD	59.0 ± 9.2	60.0 ± 9.7	59.4 ± 9.4
Male (%)	8029 (43.5)	5672 (47.3)	13701 (45.0)
Female (%)	10436 (56.5)	6308 (52.7)	16744 (55.0)
Smoking Status N (%)			
Current	1873 (10.2)	1858 (15.7)	3731 (12.4)
Former	7548 (41.2)	5158 (43.6)	12706 (42.1)
Never	8897 (48.6)	4824 (40.7)	13721 (45.5)
Education Level			
Below O level	7067 (38.3)	5585 (46.8)	12652 (41.6)
O level	2441 (13.2)	1339 (11.2)	3780 (12.4)
A level	6468 (35.0)	3806 (31.9)	10274 (33.8)
Degree	2489 (13.5)	1216 (10.2)	3705 (12.2)

An algorithm was created to relate the questionnaire to specific parkinsonian symptoms (Table 2).

**Table 2:** Summary of questions related to parkinsonian symptoms from the follow-up questionnaire

Parkinsonian symptom	Present if answered yes to these questions
Resting Tremor	Tremor or shakiness in hands? When relaxed?
Difficulty walking	Difficulty in starting to walk? Not due to arthritis?
Difficulty getting out of a chair	Difficulty getting out of a chair? Not due to arthritis?
Walking slower	Has walking become slower?
Micrographia	Has handwriting changed? Smaller?
Olfactory dysfunction	Has sense of smell changed? Less acute?

PD case ascertainment was based on self-report at baseline or 18-month follow-up questionnaire, ongoing reports of ICD-9 diagnosis of PD from computerised hospital discharge records, and ongoing searches of death certificates reports for mention of PD. Diagnostic criteria were not standardised and relied on individual clinician opinions.

### Data analyses

Analyses were conducted using Intercooled STATA 7.0. The symptom prevalence is reported for the subset of individuals who answered all symptom questions (62%), and for the total sub-cohort with missing responses as a separate response category. Chi-square tests for trend were used to examine the differences in reported parkinsonian symptom prevalence across 10-year age groups.

## Results

Of 30 447 study participants, the sub-cohort used for the present analyses included 18 465 subjects that were followed up at least through the health questionnaire eighteen months after baseline (61%). There were 8029 men (43%), mean age 59.6 ± 9.1 years and 10 436 women, mean age 58.5 ± 9.2 years. A comparison of the sub-cohort with the non-respondents indicates that the non-respondents were older, less educated, and had a higher proportion of males and smokers (Table 1). There were 39 (0.9%) men and 30 (0.3%) women with prevalent Parkinson's disease in the sub-cohort identified through medical records, death certificates and self-report. Hospital records found only one additional baseline case, and death certificates revealed two previously unreported cases.

Only 11 539 (62%) of the participants answered all questions regarding parkinsonian symptoms on the follow-up questionnaire, including 53 (30 men, 23 women) PD cases. Symptom prevalence is reported excluding individuals with incomplete responses. The prevalences are also reported for the total sub-cohort for individuals answering yes or no, or missing a response. An exploration of the pattern of missing data revealed that most individuals either answered all, none, all except one, or only one of the questions (Table 3).

**Table 3:** Patterns of missing responses according to the order of questions on the questionnaire (N = 18 465)

Order of 6 symptom questions	N (% of total)
Answered all	11539 (62.5)
Answered none	1196 (6.5)
Answered first question only	2160 (11.7)
Missing one question only	2219 (12.0)
Miscellaneous missing	1351 (7.3)

The parkinsonian symptoms were each reported by less than 10% of individuals except for slower walking, which was present in 34% of the individuals who answered all symptoms questions (Table 4). When missing answers were included as a response category, the prevalence of slower walking went down to 28%. The prevalence of slower walking which ranged from 11% to 71% in those with complete responses (8% to 56% including missing answers) from youngest to oldest age groups.

**Table 4:** The % (n) of subjects reporting symptoms and diagnosed Parkinson's disease, by 10-year age group and for PD cases

Age Group, Years (N)								
Excluding individuals with any missing symptom answers								

Symptom	40–49 (818)	50–59 (4008)	60–69 (3849)	70–79 (2803)	80+ (61)	All Ages (11 539)	P- value for age trend*	PD Cases (53)

Resting Tremor	3.9 (32)	3.6 (144)	4.3 (166)	5.4 (150)	6.6 (4)	4.3 (496)	0.0008	49.1 (26)
Difficulty Walking	4.7 (38)	5.0 (200)	3.8 (147)	3.9 (110)	6.6 (4)	4.3 (499)	0.0435	35.9 (19)
Difficulty Chair	6.6 (54)	6.4 (255)	5.7 (220)	7.2 (203)	8.2 (5)	6.4 (737)	0.3255	39.6 (21)
Walking Slower	11.3 (92)	19.6 (787)	35.9 (1383)	57.3 (1607)	70.5 (43)	33.9 (3912)	<0.0001	84.9 (45)
Micrographia	5.1 (42)	5.5 (219)	8.7 (336)	16.2 (454)	27.9 (17)	9.3 (1068)	<0.0001	73.6 (39)
Smell less acute	7.7 (63)	7.9 (315)	9.3 (357)	11.4 (319)	13.1 (8)	9.2 (1062)	<0.0001	34.0 (18)
Any of symptoms	25.7 (210)	32.6 (1306)	46.1 (1775)	65.1 (1826)	77.1 (47)	44.8 (5164)	<0.0001	96.2 (51)

Self-reported PD	0.12 (1)	0.35 (14)	0.36 (14)	0.82 (23)	1.64 (1)	0.46 (53)	0.0012	

Including Missing answers as a separate category								

Symptom	40–49 (1342)	50–59 (6341)	60–69 (6063)	70–79 (4608)	80+ (111)	All Ages (18 465)	P- value for age trend*	PD Cases (69)

Resting Tremor								
Yes	3.5 (47)	3.2 (203)	3.7 (224)	4.5 (206)	6.3 (7)	3.7 (687)	0.0026	42.0 (29)
No	84.8 (1138)	87.1 (5526)	88.8 (5385)	87.9 (4052)	83.8 (93)	87.7 (16194)		50.7 (35)
Missing	11.7 (157)	9.7 (612)	7.5 (454)	7.6 (350)	9.9 (11)	8.6 (1584)		7.3 (5)
Difficulty Walking								
Yes	3.4 (46)	4.7 (295)	3.4 (208)	3.4 (157)	6.3 (7)	3.9 (713)	0.0042	33.3 (23)
No	68.6 (921)	71.5 (4537)	76.4 (4630)	75.2 (3464)	72.1 (80)	73.8 (13632)		59.4 (41)
Missing	28.0 (375)	23.8 (1509)	20.2 (1225)	21.4 (987)	21.6 (24)	22.3 (4120)		7.3 (5)
Difficulty Chair								
Yes	5.3 (71)	5.6 (354)	5.2 (312)	6.1 (279)	6.3 (7)	5.5 (1023)	0.6787	37.7 (26)
No	66.5 (892)	69.5 (4405)	72.9 (4421)	71.2 (3281)	68.5 (76)	70.8 (13075)		58.0 (40)
Missing	28.2 (379)	24.9 (1582)	21.9 (1330)	22.7 (1048)	25.2 (28)	23.7 (4367)		4.3 (3)
Walking Slower								
Yes	8.4 (113)	16.1 (1024)	29.5 (1787)	47.0 (2165)	55.9 (62)	27.9 (5151)	<0.0001	82.6 (57)
No	61.0 (818)	56.7 (3594)	47.2 (2862)	30.7 (1417)	18.0 (20)	47.2 (8711)		14.5 (10)
Missing	30.6 (411)	27.2 (1723)	23.3 (1414)	22.3 (1026)	26.1 (29)	24.9 (4603)		2.9 (2)
Micrographia								
Yes	4.6 (62)	4.6 (294)	7.4 (446)	13.1 (604)	21.6 (24)	7.7 (1430)	<0.0001	71.0 (49)
No	65.2 (875)	69.5 (4406)	70.6 (4283)	65.4 (3012)	54.1 (60)	68.5 (12636)		24.6 (17)
Missing	30.2 (405)	25.9 (1641)	22.0 (1334)	21.5 (992)	24.3 (27)	23.8 (4399)		4.4 (3)
Smell less acute								
Yes	5.4 (73)	6.1 (388)	7.4 (450)	8.8 (407)	10.8 (12)	7.2 (1330)	<0.0001	30.4 (21)
No	61.5 (825)	66.2 (4199)	68.7 (4164)	68.9 (3175)	65. 8 (73)	67.4 (12436)		56.5 (39)
Missing	33.1 (444)	27.7 (1754)	23.9 (1449)	22.3 (1026)	23.4 (26)	25.4 (4699)		13.1 (9)
Any of symptoms								
Yes	15.7 (210)	20.6 (1306)	29.3 (1775)	39.6 (1826)	42.3 (47)	28.0 (5164)	<0.0001	73.9 (51)
No	45.3 (608)	42.6 (2702)	34.2 (2074)	21.2 (977)	12.6 (14)	34.5 (6375)		2.9 (2)
Missing	39.0 (524)	36.8 (2333)	36.5 (2214)	39.2 (1805)	45. 1 (50)	37.5 (6926)		23.2 (16)

Self-reported PD	0.07 (1)	0.25 (16)	0.31 (19)	0.69 (32)	0.90 (1)	0.37 (69)	<0.0001	

*Calculated using chi-square for trend for each individual symptom across the age groups.

The proportion of subjects reporting each symptom significantly increased with age, with the exception of difficulty getting out of a chair. Difficulty walking was relatively constant across the age groups, until the age of 80 when there was a sharp increase. The age trends were similar for males and females, with the exception of difficulty getting out of a chair, which was age-dependent for males but less so for females (Results not shown). The percentage of PD cases reporting each parkinsonian symptom ranged from 34% to 85% (30% to 83% including missing responses), and as expected all symptoms were reported in proportionally more PD than non-PD subjects (Table 4).

The proportion of individuals self-reporting PD increased with age. The prevalence of PD in the individuals answering all symptoms questions was 0.46%, and 0.37% in the total sub-cohort (Table 4). Anti-parkinsonian medication use was recorded according to the British National Formulary. Nineteen identified PD cases were using at least one of selegiline, pergolide, and levodopa. The use of bromocriptine and procyclidine, which are not specific treatments for PD, was reported by nine individuals without self-reported PD.

The sensitivity and specificity for each symptom question as well as cumulative numbers of symptoms questions are reported in Table 5. The question with the highest sensitivity (85%) asked about walking slower, but the specificity was very low (66%). If a positive response to any of the six symptoms questions was defined as screening positive, then the sensitivity was 96% and specificity was 56%, but if slower walking was excluded then sensitivity was 91% and specificity 76%.

**Table 5:** The sensitivity and specificity of the self-reported questionnaire for PD identified by self-report and routine records (N = 11 539)

	TP	FN	Sensitivity	TN	FP	Specificity
Rest Tremor	26	27	49.1	11016	470	95.9
Difficulty Walking	19	34	35.9	11006	480	95.8
Difficulty Chair	21	32	39.6	10770	716	93.8
Walking Slower	45	8	84.9	7619	3867	66.3
Micrographia	39	14	73.6	10457	1029	91.0
Olfactory dysfunction	18	35	34.0	10442	1044	90.9
Any of 6 symptoms	51	2	96.0	6373	5113	55.5
Any of symptoms except Walking slower	48	5	90.6	8684	2802	75.6
≥2 symptoms	46	7	86.8	9705	1781	84.5
≥3 symptoms	38	15	71.7	10930	556	95.2
≥4 symptoms	22	31	41.5	11363	123	98.9
≥5 symptoms	11	42	20.8	11455	31	99.7

TP = True Positive; FN = False Negative; TN = True Negative; FP = False Positive

The prevalence of parkinsonian symptoms in the total sub-cohort was similar for the age groups < 65 and 65+ years, with the exception of micrographia and walking slower, which were reported twice as often by the older age group (Table 6).

**Table 6:** Prevalence (%) of parkinsonian symptoms in elderly populations, with and without parkinsonism, from screening questionnaire studies and current* study.

Total Populations						
	Current		Mutch [4]	Pramstaller [5]	Rocca [6]	Teresi [7]
Number of Subjects	6771	4768	87	36	16	164
Age (Years)	<65	>65	71–76	40+	62–96	65+

Symptom						
Arms or legs ever shake?			5.0	35.0	6.2	
Resting Tremor	3.8	5.0				9.9

Shuffle feet			3.0	8.3	31.2	10.1
Feet suddenly freeze in doorways				2.8	6.2	
Poor balance			10.0	27.8	37.5	
Difficulty Walking	4.7	3.8				
Walking Slower	22.1	50.7				3.8#

Trouble rising from chair	6.4	6.4	13.0	13.9	37.5	21.4
Micrographia	6.0	13.9	14.0	5.6	12.5	13.2
Olfactory dysfunction	7.7	11.3				

Patients with Parkinsonism						

	Current	Duarte [28]	Meneghini [31]	Mutch [4]	Pramstaller [5]	Rocca [6]
Number of Subjects	53	36	21	35	40	37
Age (Years)	46–81	30–89	40+	57–89	40+	58–94

Symptom						
Arms or legs ever shake?		89.1	76.2	77.0	65.0	64.9
Resting Tremor	49.1					

Shuffle feet		72.9		51.0	82.5	67.6
Feet suddenly freeze in doorways		51.3			52.5	56.8
Poor balance		83.7		69.0	75.0	81.1
Difficulty Walking	35.9					
Walking Slower	84.9					

Trouble rising from chair	39.6	59.0		66.0	67.5	75.7
Micrographia	73.6	64.8		74.0	90.0	73.0
Olfactory dysfunction	34.0					

*Current study including only the individuals who answered all symptoms questions (N = 11 539)

# Interviewer observation

## Discussion

The purpose of this paper was not to validate a possible screening questionnaire, but to report the responses of the EPIC cohort to questions about the prevalence of various parkinsonian symptoms, and examine how these relate to age and self-reported PD. Similar questionnaires have been validated and various cut-off points used to identify individuals for further examination in a two-step screening process [5,6,21-32]. However, most of these did not report the responses for each question, but instead reported the sensitivity and specificity for a specific chosen cut-off point. Sensitivity was often quite high, however the positive predictive value for Parkinson's disease after screening was often low. Sensitivity and specificity are reported for the questionnaire, with self-reported PD as the disease outcome. Neurological examinations were not conducted to validate diagnoses or to identify de novo cases.

### Study population

The current study population was a sub-cohort that completed the first follow-up questionnaire of the EPIC-Norfolk study. The population was restricted by age at baseline (45–74 years) and was ethnically homogeneous. The consequence of the homogeneous ethnicity and limited study area is that the results of this study may only be generalizable to Caucasians of similar socio-economic status and environmental conditions. Ethnic and geographical differences must be considered as possible explanations for differences in prevalence between studies, once methodological differences are accounted for. We have combined men and women for the analyses because there were few PD cases, and age was considered a more pertinent variable.

Selection bias was introduced at the original recruitment of participants for EPIC and in the exclusion of participants who had not answered the follow-up health questionnaire. Individuals who were ill at baseline were excluded from the study, and those who became ill after the baseline exam would be more likely to have dropped out of the study by the questionnaire at eighteen months. The prevalence estimates from this study are expected to be underestimates of the true measures in the Norfolk population because the sub-cohort is healthier than the general population and the original study population. However, the expected number of 72 cases, as calculated by indirect standardization using a prevalence study carried out in general practices in London, is similar to the 69 prevalent cases identified in the EPIC sub-cohort [33].

A further attrition of participants occurred because 38% of the sub-cohort did not respond to one or more of the symptom questions. The number of missing responses was associated with age, education, and smoking. All analyses of symptom prevalence were conducted with the subset of 62% of individuals who answered all questions, and repeated assuming that missing answers indicated the absence of the symptom.

Subjects missing the date of the second health examination were excluded to avoid an inconsistent denominator for analyses. Among the excluded participants were less than 3% of the sub-cohort, but this included 4 PD cases.

### Methods

The questions regarding the presence of parkinsonian symptoms were subject to participant interpretation and relied on complete answers in the follow-up health questionnaire. The patterns of missing data according to the order of the questions in the questionnaire showed that most individuals answered all, none, all except one, or only one of the questions. It is difficult to hypothesize why individuals did not answer certain questions. Those answering none of the questions or only the first symptoms question could be due to the format of the questionnaire. The first part of the question asks about the diagnosis of PD, followed by the symptoms questions. It might improve the response if the symptoms questions were asked first, so those without PD diagnoses would not assume that the following questions did not apply to them.

It was not possible to conduct neurological examinations for all cohort participants, as Parkinson's disease is a secondary outcome for the EPIC-Norfolk study. However, a review reports that self-rated health status has been shown in many studies to be an accurate predictor of mortality, even better than medical records [34]. Self-rated health and self-reported parkinsonian symptoms are indeed two different measures, however the review emphasizes the value of self-report, even with its limitations. A cross-sectional study reported correlation between mild parkinsonian symptoms, measured with a shortened version of the UPDRS, and both self-reported measured of function and performance-based test scores [35]. Some literature comparing self-report with neurological examination, although not specifically for PD, offers further support [36].

The PD ascertainment methods were incomplete and relied heavily on the accuracy of self-report. PD self-report can be assumed to be relatively accurate because it is a serious condition without a negative social stigma [37-40]. However, all ascertainment methods relied on a previous diagnosis of PD by medical practitioners, when it is known that misdiagnosis in the community setting can be greater than 25%, and underdiagnosis more than 20% [33,41]. Another possible cause for underreporting was that the baseline questionnaire did not specifically ask about past diagnosis of PD, so the participant had to write the disease in a part of the form to list 'other serious illnesses.' Underascertainment and underdiagnosis would dilute the observed difference in reported symptoms between PD cases and controls because there would be unidentified cases in the control group. However, the control group is relatively large so the effect would be minimal.

Sensitivity and specificity were calculated for each question and combinations of questions, however the outcome measure was self-reported PD. In the absence of a true gold standard pathological marker for PD, a thorough neurological examination by a movement disorders specialist would be preferred. The measures in this study show how well the self-reported symptom questions relate to self-reported PD. De novo PD cases would not be included in the cases, therefore the true sensitivity and specificity of the questionnaire are unknown. The values do give a good indication of which questions might not be useful for distinguishing cases from controls, such as slower walking.

### Comparisons and conclusions

Although this study was not an evaluation of a screening instrument, the studies reporting the responses to similar questionnaires were all either for screening validation or prevalence surveys [4-7,28,31]. Positive responses are compared between the individuals in the sub-cohort who answered all symptoms questions, for above and below 65 years of age, and the unaffected controls from other studies. The symptom responses are also compared between PD cases (Table 6). The proportion of individuals with complete responses reporting individual parkinsonian symptoms in the sub-cohort was approximately 4–10% for resting tremor, difficulty walking, difficulty getting out of a chair, micrographia, and olfactory dysfunction. In addition, five studies conducted neurological examinations to determine the presence of neurological signs in elderly populations [1,8-11]. The neurological signs tremor, gait disturbance, and shuffling feet correspond with some of the previous questionnaire symptoms, but only tremor was included in the current study questionnaire.

If resting tremor and shaking of arms or legs are considered to be the same symptom, most of the questionnaire studies report prevalence less than 10% except for the 35% reported by a relatively young control group in Pramstaller et al [5]. There is a large range across the neurological examination from 1% to 30%, which is not explained by age. A number of differences could explain the variation including the differences in study populations and assessment methods. Resting tremor was reported by 42% of the cases, which is low compared to the 75–90% reported in several other studies [42-45]. Some of the studies asked about tremor or shaking without specifying that it was occurring at rest. If the tremor question alone is used then 43 (81.1%) of PD cases and 888 (8%) of individuals with complete symptoms responses answered positively.

Walking slower was the most commonly reported symptom in cases and in all EPIC subjects who responded to all questions. The only other study to report on this symptom used interviewer observation rather than a questionnaire for assessment, therefore the much lower prevalence in that study is not truly comparable.

Trouble rising from a chair was reported less frequently by EPIC participants with complete responses compared with other questionnaires. The same was true for PD cases. The presence of micrographia within EPIC corresponded with other questionnaires for the two age groups, below and above 65 years, and for PD patients. Difficulty walking was not specifically asked on comparison questionnaires, although other symptoms related to walking are reported (Table 6). Olfactory dysfunction was only reported by EPIC.

The lower prevalence of parkinsonian symptoms in the EPIC sub-cohort compared to the previous studies could be explained by selection bias for a healthy cohort, although the observed prevalence of PD from self-report was close to the expected prevalence of PD [33]. The lower prevalence of symptoms in EPIC PD cases compared to cases from other studies could be due to less severe or more recently diagnosed disease in the EPIC participants, although dates of diagnosis are not available at this time.

The age division of EPIC participants showed that only walking slower and micrographic were much more frequent in individuals above 65 years. The other symptoms were similar in both groups, which is unexpected, however the questions may not be specific to the symptom of interest since the question is dependent on individual interpretation.

Despite the limitations of the study, there were important findings concerning the reported prevalence of parkinsonian symptoms in the sub-cohort and stratified by self-reported PD status. The results are not unexpected but it is useful to support evidence that individual and cumulative self-reported parkinsonian symptoms are strongly associated with self-reported PD diagnosis and increasing age. The data also provide information regarding the prevalence of symptoms in a younger population of adults than previously reported in the literature.

## Competing interests

The author(s) declare that they have no competing interests.

## Authors' contributions

LI participated in the design of the study, carried out statistical analyses, and drafted the manuscript. KK, RL, SB, AW, and ND conceived of the study, and participated in its design and coordination. CB participated in the design of the study, data analysis methods, and critical revision of the manuscript. All authors read and approved the final manuscript.

## Pre-publication history

The pre-publication history for this paper can be accessed here:


